# Identification of Candidate Genes Associated with Leaf Senescence in Cultivated Sunflower (*Helianthus annuus L.*)

**DOI:** 10.1371/journal.pone.0104379

**Published:** 2014-08-11

**Authors:** Sebastian Moschen, Sofia Bengoa Luoni, Norma B. Paniego, H. Esteban Hopp, Guillermo A. A. Dosio, Paula Fernandez, Ruth A. Heinz

**Affiliations:** 1 Instituto de Biotecnología, Centro de Investigaciones en Ciencias Agronómicas y Veterinarias, Instituto Nacional de Tecnología Agropecuaria, Hurlingham, Buenos Aires, Argentina; 2 Consejo Nacional de Investigaciones Científicas y Técnicas, Ciudad Autónoma de Buenos Aires, Argentina; 3 Escuela de Ciencia y Tecnología, Universidad Nacional de San Martín, San Martín, Buenos Aires, Argentina; 4 Facultad de Ciencias Exactas y Naturales, Universidad de Buenos Aires, Ciudad Autónoma de Buenos Aires, Argentina; 5 Laboratorio de Fisiología Vegetal, Unidad Integrada Universidad Nacional de Mar del Plata, Estación Experimental Agropecuaria INTA Balcarce, Balcarce, Buenos Aires, Argentina; University of North Carolina at Charlotte, United States of America

## Abstract

Cultivated sunflower (*Helianthus annuus L.*), an important source of edible vegetable oil, shows rapid onset of senescence, which limits production by reducing photosynthetic capacity under specific growing conditions. Carbon for grain filling depends strongly on light interception by green leaf area, which diminishes during grain filling due to leaf senescence. Transcription factors (TFs) regulate the progression of leaf senescence in plants and have been well explored in model systems, but information for many agronomic crops remains limited. Here, we characterize the expression profiles of a set of putative senescence associated genes (SAGs) identified by a candidate gene approach and sunflower microarray expression studies. We examined a time course of sunflower leaves undergoing natural senescence and used quantitative PCR (qPCR) to measure the expression of 11 candidate genes representing the NAC, WRKY, MYB and NF-Y TF families. In addition, we measured physiological parameters such as chlorophyll, total soluble sugars and nitrogen content. The expression of *Ha-NAC01, Ha-NAC03, Ha-NAC04, Ha-NAC05* and *Ha-MYB01* TFs increased before the remobilization rate increased and therefore, before the appearance of the first physiological symptoms of senescence, whereas *Ha-NAC02* expression decreased. In addition, we also examined the trifurcate feed-forward pathway (involving *ORE1*, *miR164*, and *ETHYLENE INSENSITIVE 2*) previously reported for Arabidopsis. We measured transcription of *Ha-NAC01* (the sunflower homolog of *ORE1*) and *Ha-EIN2*, along with the levels of *miR164*, in two leaves from different stem positions, and identified differences in transcription between basal and upper leaves. Interestingly, *Ha-NAC01* and *Ha-EIN2* transcription profiles showed an earlier up-regulation in upper leaves of plants close to maturity, compared with basal leaves of plants at pre-anthesis stages. These results suggest that the *H. annuus* TFs characterized in this work could play important roles as potential triggers of leaf senescence and thus can be considered putative candidate genes for senescence in sunflower.

## Introduction

As the last stage of leaf development, the genetically determined and highly ordered process of senescence involves characteristic changes in gene expression that result in decreased photosynthetic activity, active degradation of cellular structures, nutrient recycling, lipid peroxidation and, ultimately, cell death [1], [2]. Multiple variables control the complex mechanism of senescence; these genetic and environmental variables have a strong effect on crop yield [3]. Annual plants, such as grain and oil crops, undergo visible senescence towards the end of the reproductive stage, accompanied by nutrient remobilization from leaves to developing seeds [4]. In monocarpic species, the development of the reproductive structure controls leaf senescence [5]. Prematurely induced senescence, caused by biotic or abiotic stress, can reduce crop yield. Thus, leaf senescence has an economic impact, affecting the gap between potential and real yields.

Sunflower (*Helianthus annuus L*.) is the third most important source of edible vegetable oil worldwide, and the second in Argentina. It also provides an important source of biodiesel [6] (Sunflower Statistics NSA 2007–2009, USA) [7]. Recent work has produced some genomic information for this crop [8], but the complete genome sequence remains unavailable. However, functional genomics tools for cultivated sunflower have been developed, including transcriptional and metabolic profiling tools [9]–[21].

In sunflower, adverse environmental conditions and foliar diseases abruptly trigger senescence [22], resulting in seriously limited production. In different crops, including sunflower, a delay in leaf senescence has an important impact on yield, by maintaining photosynthetic leaf area during the reproductive stage [23]–[26]. The maintenance of functional leaves for longer periods could increase the intercepted radiation and thus favour seed weight and oil content during the grain filling period [27], [28].

Sunflower is an annual, monocarpic species in which the reproductive organs exert a strong control on leaf senescence and nutrient remobilization, and final grain mass is affected by the source:sink ratio [29]. However, the age of a leaf, and its position on the stem affect the triggering of senescence and the rate of the remobilization of nutrients [30]. Given the high extinction coefficient of light in the canopy for sunflower, shading of lower leaves affects not only the amount of incident radiation, but also the quality of the light, thereby causing senescence [31].

Two main stages can be distinguished in the senescence process: an initial stage involving up- or down-regulation of expression of a set of genes involved in nutrient export and degradation of cell structures, and a second stage involving progression of senescence, which proceeds at different rates under different conditions. The first stage of senescence can be assessed by measuring gene expression and the second stage can be assessed by measuring physiological traits. For example, chlorophyll content, the most commonly measured parameter, is directly associated with turnover of CO_2_-fixing Rubisco [30], [32]. However, other physiological parameters, such as the drop in soluble sugars, can also be measured to evaluate the progression of senescence [33], [34]. Nitrogen content represents an important leaf senescence-associated variable, given its central role in key cellular processes such as photosynthesis. Previous studies examined several physiological parameters related to leaf senescence in sunflower [9], [35]–[37], but the relationship of these parameters to gene expression, particularly the induction of transcription factors at an early stage of leaf development, has not been reported yet.

NAC transcription factors related to senescence have been identified in model (Arabidopsis thaliana, Medicago sativa, Orzya sativa L., Brassina napus) and non-model species (Chrysanthemum lavandulifolium, Gossipium hirsutum L., Malus domestica, Solanum tuberosum L., Triticum aestivum) and described as relevant players in the regulation of leaf senescence, particularly related to programmed cell death [38]–[56]. In Arabidopsis thaliana, the expression of a NAC gene family member, ORE1, occurs under the control of the ethylene signaling pathway gene EIN2 (ETHYLENE INSENSITIVE2) [45]. EIN2 encodes a central protein of ethylene signaling that is located in the endoplasmic reticulum membrane [57], [58] and is down-regulated by miR164. In young leaves, which have high levels of miR164, ORE1 expression remains low, but during the leaf aging process, as miR164 expression gradually decreases, ORE1 expression increases [38]. Despite evidence of ORE1 function in senescence, our knowledge about the possible targets of this transcription factor remains limited. Recent work identified the preferred binding sequence of ORE1, which directly activates BIFUNCTIONAL NUCLEASE1 (BFN1) [43].

Previous work on leaf senescence in sunflower identified *Hahb-4*, a transcription factor related to the ethylene signaling pathway [59]. Ethylene positively regulates *Hahb-4* during normal leaf senescence; once induced, Hahb-4 negatively regulates the biosynthesis of ethylene and the expression of genes related to this signaling pathway.

In this work, we evaluated the transcription profiles of NAC, AP2/EREBP, WRKY, MYB and NF-Y family TF as potential candidate genes, as well as *miR164*, in sunflower leaves at different developmental stages during natural senescence. Concomitantly, we also measured physiological parameters such as chlorophyll, total soluble sugars and nitrogen content, to assess the triggering time of the different functional variables along the onset and evolution of senescence in sunflower, a relevant oil crop with limited available genomic information.

## Materials and Methods

### Plant material and experimental conditions

A field experiment was carried out at the INTA Balcarce Experimental Station (37°45′ S, 58°18′ W) during the 2010/11 growing season. Sunflower hybrid VDH 487 (Advanta Seeds) was sown at a 7.2 plants/m^2^. Emergence occurred 10 days later. Diseases, weeds and insects were adequately controlled. Soil fertility assured maximum yields under non-limiting water conditions. Soil volumetric humidity was measured periodically using the time domain reflectometry technique (Trase System, Model 6050X1, Soil moisture Equipment Corp., Santa Barbara, CA, USA). Soil water was maintained by irrigation above 50% of volumetric humidity in the first 0.60 m of soil during the entire growing season.

Time was expressed on a thermal time basis by daily integration of air temperature with a threshold temperature of 6°C and with plant emergence as thermal time origin [60].

The experiment was conducted as a randomized complete block design with three replicates (plant–plots). Each biological replicate consisted of three randomly selected plants from each plot. Both molecular and physiological measurements were performed in tissue obtained from leaves 10 and 20. Both leaves were sampled after they reached about 15 cm width and with an accumulation of degree days from emergence (°Cd) between 432 to 861 and 670 to 1180, for leaves 10 and 20 respectively.

### Chlorophyll measurements

Chlorophyll content of the sampled leaves was measured by chemical extraction with N, N dimethylformamide [61]. Two 0.5 cm-diameter discs were taken from the base of each sampled leaf. A total of 6 disks for each biological replicate (3 plants sampled in each plot) were dried with tissue paper and incubated in vials containing 6 ml of N, N dimethylformamide overnight at room temperature in darkness. Absorbance of each sample was measured using a spectrophotometer (Spectronic 20, Bausch & Lomb, Bausch & Lomb Place, Rochester, New York, USA). Chlorophyll content was calculated as:

Chlorophyll (mg.l^−1^) = 17.9 * abs (647)+8.08 * abs (664)

Chlorophyll (mg.cm^−2^) = Cl (mg.l^−1^)/1.1775 cm^2^

Where: *abs (647)* = absorbance at 647 nm, corresponding to the maximum absorption peak of chlorophyll B; *abs (664)* =  absorbance at 664 nm, corresponding to the maximum absorption peak of chlorophyll A and the values 17.9 and 8.08 are the extinction coefficients of chlorophylls A and B, respectively [61]

### Total soluble carbohydrates (TSC)

Three 1.2 cm-diameter discs were taken from the base of each leaf sampled. A total of 9 disks for each biological replicate (3 plants sampled in each plot) were weighed and dried at 60°C for 48 hours. Five ml of distilled water was added to a test tube containing 50 mg of dry tissue. Samples were incubated in a water bath at 100°C for 10 min and centrifuged at 2500 rpm for 5 min; this step was repeated three times. A 100 µl volume of the supernatant was placed in a test tube and distilled water was added to a volume of 1 ml. TSC were quantified by a colorimetric method using a phenol and sulfuric acid reaction [62]. One volume of phenol 5% and five volumes of sulfuric acid were added to the solution and incubated for 30 min at 25°C. After a color reaction with a mixture of phenol / sulfuric acid, optical density was measured in a spectrophotometer at 490 nm (Spectronic 20, Bausch & Lomb, Rochester, New York, USA). Total soluble carbohydrates were deduced from a standard curve constructed with glucose standard solutions (0–15 µg.µl^−1^).

### Total Nitrogen (%)

The percentage of total nitrogen was measured according to the Dumas method from 60 mg of dry tissue [63]. Briefly, the Dumas method consists of a dry combustion at 950°C, using oxygen as the combustion accelerator. Combustion products (H_2_O, NO, N_2_) are filtered and dried. NO is reduced to N_2_ by copper, and is swept by helium gas to a thermal conductivity cell where the concentration is measured (TrunSpec CN, Leco, Michigan, USA).

### RNA isolation and quality controls

Total RNA isolation was performed on healthy leaf samples starting from 430°Cd after emergence in order to assure RNA integrity. Samples were immediately frozen in liquid nitrogen and saved at −80°C until processing. High quality total RNA was isolated from 100 mg of frozen tissue using TRIzol, following the manufactureŕs instructions (Invitrogen, Argentina). Genomic DNA was eliminated after treatment with DNase I for 20 min at room temperature using DNase I (Invitrogen, Argentina).

RNA concentration was measured using a Nanodrop ND-1000 spectrophotometer (NanoDrop Technologies, Wilmington, Delaware USA). Purity and integrity of total RNA was determined by 260/280 nm ratio and the integrity was checked by electrophoresis in 1.5% agarose gel.

### Selection of Transcription Factors

Eleven transcription factors were selected by a literature search for putative orthologs of candidate genes associated with leaf senescence that have been reported in model species, as well as TFs identified based on differential expression levels, from a customized 4×44 K microarray analysis conducted on the Agilent platform [15], in which leaf samples at different development stages and growing conditions were assessed (Table S1 and S2) (unpublished data). Statistical analysis was performed using in house routines to fit, gene by gene, a linear mixed-effects model. The Sunflower Custom Oligo Microarray includes 4 arrays per chip; therefore, the chip effect (incomplete block) was included as a random effect. The set of routines mentioned above were based on the *lme* function of the *nlme* library of R [64] implemented in InfoStat statistical software [65]. Differential gene expression analysis was carried out using the limma package. Gene set analysis was carried out according to the Gene Ontology terms using FatiScan [66] integrated in the Babelomics suite [67].

### Primer design for Reference Genes and SAGs

Different reference genes were assessed in a previous gene expression study in sunflower leaf senescence [68]. In this study, Elongation Factor 1-α (*Ha-EF-1α*) was selected as a reference gene, as it showed stable expression in the different samples for both leaves.

Sunflower Unigene Resource (SUR v1.0) database available at http://atgc-sur.inta.gob.ar// was used to search candidate TFs [15].

Specific primer pairs for qPCR were designed based on selected sequences using Primer3 software [69] with the default parameters (Table 1).

**Table 1:** Primer sequences for qPCR analysis.

Sunflower TF	Primer sequence Left	Primer sequence Right
*Ha-NAC01*	AAGAAGTACCCGACCGGATT	TCACCCAATTCGTCTTTTCC
*Ha-NAC02*	GACTTCCGTTGATCCGACAT	AATGGGTCGGTTGTGCTTAG
*Ha-NAC03*	ATTTCTGGCGGTCAAACAAC	CCGGTCTTGTATCTCGGGTA
*Ha-NAC04*	TGGTGTGAAGAAGGCACTTG	TTCGACACAAAACCCAATCA
*Ha-NAC05*	ATGTTTGGCGAGAAGGAATG	TCCTTTCGGAGCTTTACCAA
*Ha-WRKY01*	CATAATGCCCCATTCAATCC	CATTTTGCTTGGTTGGAGGT
*Ha-MYB01*	TACTTGCCCGAAAATCCAGT	ATTGCTCGTCCAATCAATCC
*Ha-MYB02*	GAAGTAATGCCCCTGGATCA	TTCTTGCCATTGAACTGCTG
*Ha-RAV01*	CCTCATCGCGATACAAAGGT	GCCTTTGCAGCTTCATCTTC
*Ha-NF-YB3*	ACGTCAGGGTGGTGAAAAAG	ATAACCCGAACCGATTTTCC
*Ha-EIN2*	AGCTGGCGTTCTCTAAACCA	TGCAATCTCCACATCCTTCA
*Ha-CAB2*	TTGATCCATGCACAAAGCAT	AGCTACCACCGGGGTAAAGT
*Ha-EF-1α*	ACCAAATCAATGAGCCCAAG	GAGACTCGTGGTGCATCTCA

### Quantitative RT-PCR analysis

For each sample, 500 ng DNase treated RNA was reverse-transcribed using Superscript III first strand synthesis system (Invitrogen, USA) and random hexamer primers according to the manufacturer's instructions. qPCR was carried out in a 25-µl reaction mix containing 200 nM of each primer, 1 µl of cDNA sample and FastStart Universal SYBR Green Master (Roche Applied Science). Negative controls (no RT added and non-template control) were incorporated in the assays. qPCRs were performed using a 96-well plate thermocycler (ABI Prism 7000 Sequence Detection System and software, PE Applied Biosystems, USA). The thermal profile was set to 95°C for 10 min, and 40 cycles of 95°C for 15 s, and hybridization temperature for 1 min. Amplicon specificity was verified by melting curve analysis (60 to 95°C) after 40 PCR cycles. The qPCR assay was carried out using three biological replicates for each condition, and two technical replicates from two independent cDNA synthesis reactions.

**Figure 1:**
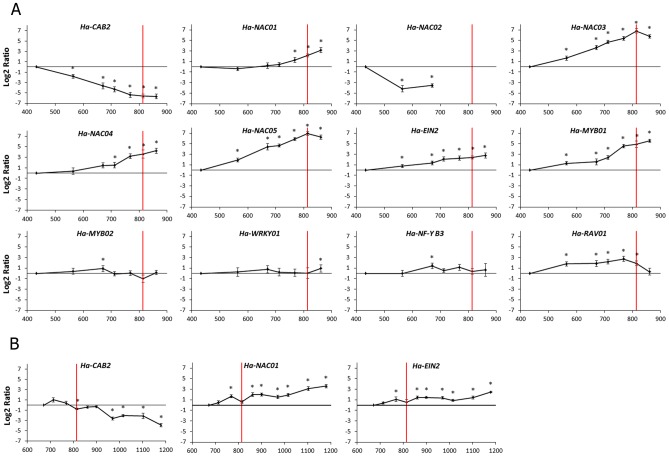
qPCR expression data. (A) Leaf 10. (B) Leaf 20. Relative transcript level for each candidate gene at different sampled points determined by qPCR. The x –axis showed the thermal time after emergence (°Cd). Asterisks indicate significant difference between each sampling in relation to first sampling point (S-1) and reference genes (p-value<0.05). The red line indicates anthesis time. Error bars correspond to standard errors.

A qPCR assay was performed to quantify the expression of candidate genes in leaves 10 and 20 (numbered from the bottom to the top of the plant) at different sampling times during plant development. Expression of these genes was estimated in relation to Elongation Factor -1α (*EF-1α*) previously selected as a reference gene [68]. Amplification efficiencies and Ct values were determined for each gene and each tested condition, with the slope of a linear regression model using the LinRegPCR [70]. These profiles were estimated in relation to first sampling (S-1) and reference genes using fgStatistic software (Figure 1A and B) [71], based on previously published algorithms [72].

### miRNA Northern blot

High quality total RNA was isolated from leaves by using TRIzol, following the manufactureŕs instructions (Invitrogen, Argentina), repeating the chloroform extraction two times. Fifteen micrograms of RNA was resolved in 17% polyacrylamide gels containing 7 M urea. After electrophoresis, RNA was blotted to GeneScreen Plus membrane (PerkinElmer Life Science, Waltham, MA). Sunflower microRNA164 probe (5′TTCATGTGCCCTGCTTCTCCA3′) [73] was end-labelled using 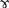
ATP and T4 Polynucleotide Kinase. The labelled probe was purified using the QIAquick Nucleotide Removal kit (QIAGEN, Argentina). The eluted radiolabeled oligonucleotide was incubated with the membrane in 5× SSC, 7% SDS, 20 nM Na_2_HPO_4_ (pH7.2), 2× Denhardt's solution and 1 mg of sheared salmon sperm DNA at 50°C overnight.

The membrane was washed two times with washing solution containing 3× SSC, 5% SDS, 25 nM NaH_2_PO_4_ (pH7.5), 10× Denhardt's solution for 15 minutes and exposed overnight. The intensity of each band was quantified using a Typhoon Trio (Amersham Biosciences, Piscataway, NJ). Radioactivity intensity of each band was normalized based on ethidium bromide rRNA labelled loaded in each well.

## Results

### Measurement of Physiological Parameters

**Figure 2:**
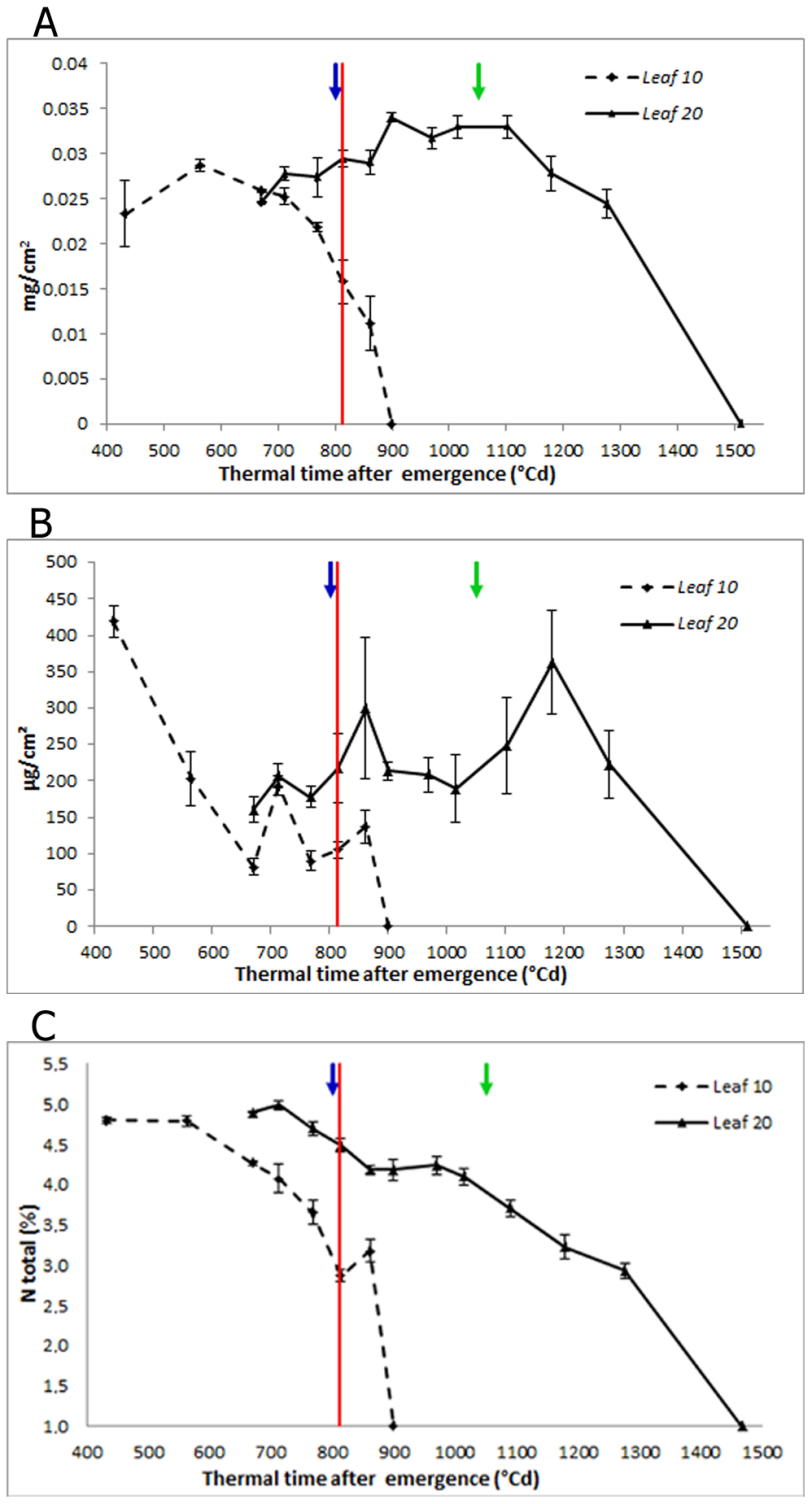
Physiological measurements of the progression of senescence. (A) Chlorophyll content in mg.cm^−2^. (B) Total soluble carbohydrates in µg/cm^2^ and (C) Total nitrogen percentage. The red line indicates anthesis time. Blue and green arrows show the date when the *Ha-NAC01* expression ratio reaches a value of 4 (Log2 ratio = 2) relative to first sampling point in leaf 10 and 20, respectively, determined by qPCR (*Ha-EF1α* as RG). Error bars correspond to standard errors.

We assessed the progress of senescence by measuring changes in chlorophyll content, nitrogen and soluble carbohydrates. These three physiological variables showed similar profiles in leaves 10 and 20, numbered from the bottom of the plant (Figure 2A, B and C).

The maximum chlorophyll content in leaf 10 reached 0.03 mg.cm^−2^ at more than 200°Cd before flowering time. From 700°Cd after emergence, chlorophyll began to decline until it became zero, at close to 900°Cd (Figure 2A). In leaf 20, the maximum chlorophyll content was slightly higher than in leaf 10 (close to 0.035 mg.cm^−2^), and the maximum was reached 900°Cd after emergence, about 100°Cd after flowering, when leaf 10 had already senesced. In leaf 20, chlorophyll began to decline at 1,100°Cd, and leaf 20 senesced 400°Cd later (Figure 2A). Leaf 20 had a longer lifespan; despite having been initiated at the apex 115°Cd later (data not shown), its active lifespan was lengthened by 600°Cd compared to leaf 10.

After a high initial value above 400 µg.cm^−^^2^, total soluble carbohydrates in leaf 10 decreased to between 100 and 150 µg.cm^−^^2^, from 600 to 850°Cd after emergence, just after flowering. Then, they dropped abruptly until leaf death at 900°Cd (Figure 2B). In leaf 20, carbohydrates remained relatively stable between 200 and 250 µg.cm^−^^2^ from 650 to 1,000°Cd after emergence. Nearly 1,100°Cd after emergence, soluble carbohydrates increased up to 350 µg.cm^−^^2^, and thereafter started to drop at 400°Cd after flowering, reaching zero at senescence, around 1,500°Cd (Figure 2B).

The maximum N (%) content was close to 5% in both leaves (Figure 2C). In leaf 10, it started to decline at 600°Cd and in leaf 20 at 700°Cd after emergence. In both leaves the content of N started to decline before flowering; at flowering, the N content in leaf 10 had already diminished to 50% of its maximum content, and the N content in leaf 20 had decreased to 80% of maximum. Furthermore, the decline was markedly slower in leaf 20 than in leaf 10 (Figure 2C).

### Measurement of Transcription Factor Gene Expression

We used BLASTX [74] to identify putative TFs highly similar to Arabidopsis SAGs. Searches of the *Sunflower Unigene Resource (SUR v1.0)* [15] identified 42 genes with a significant score. Out of these, we selected 11 TFs by a candidate gene strategy based on literature searches in model species and on gene expression levels measured in a sunflower microarray analysis (unpublished data). The selected genes included NAC, WRKY, MYB, RAV, NF-Y, and AP2 TFs (Table S1 and S2).

**Figure 3:**
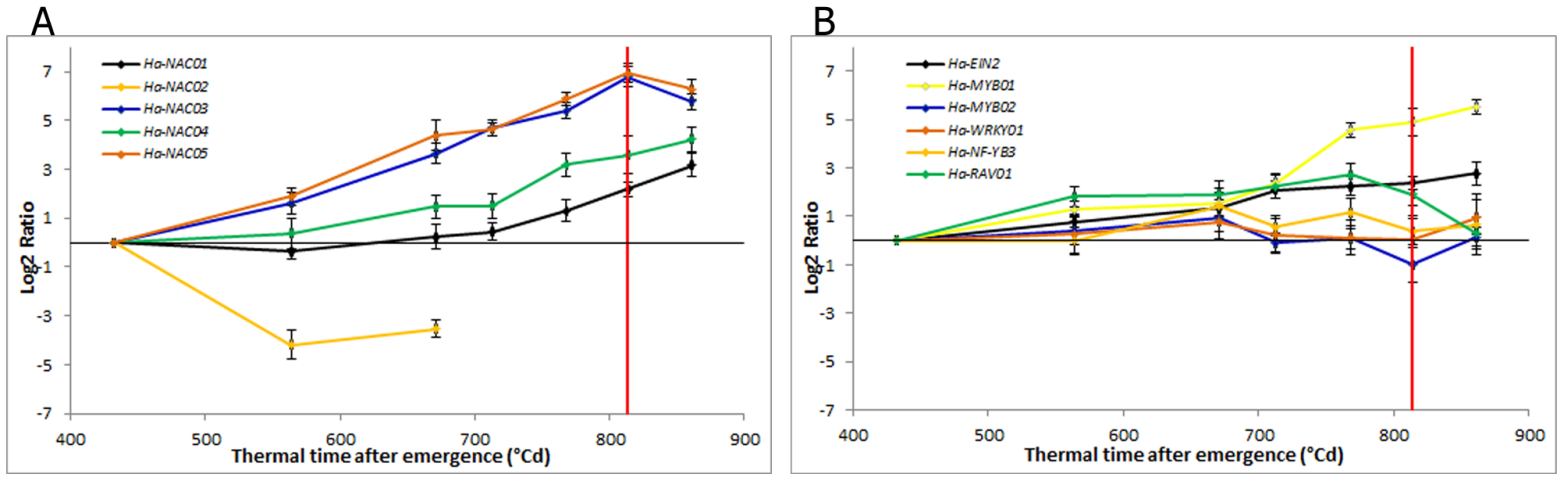
Expression profiles of transcription factors. (A) NAC TFs relative transcript level at each sampled point in relation to the level at the first sampling point determined by qPCR in leaf 10 (*Ha-EF1α* as RG). (B) AP2/EREBP, MYB, WRKY, NF-Y and RAV TFs relative transcript level at each sampled point in relation to the level at the first sampling point determined by qPCR in leaf 10 (*Ha*-*EF1α* as RG). The red line indicates anthesis time. Error bars correspond to standard errors.

NAC TF transcript levels increased significantly during leaf development (Figure 3A). All of the NAC TFs tested showed an up-regulation at early stages of leaf development, except *Ha-NAC02*, which showed down-regulated expression at an early stage. *Ha-NAC01* and *Ha-NAC04* transcript levels gradually increased from emergence, with high transcript levels in later developmental phases, close to anthesis. Moreover, *Ha-NAC03* and *Ha-NAC05* transcript levels increased at an early stage of leaf development, with continued increases in expression until anthesis, when they reached their highest levels. Transcript levels of *Ha-EIN2* gradually increased at an early stage and then showed uniform expression through leaf development (Figure 3B). In contrast, *Ha-MYB01* transcript levels gradually increased in early stages, then strongly increased from 700°Cd until the end of leaf development. *Ha-RAV01* transcript levels also gradually increased up to 750°Cd and then declined until the last stage of development. Transcript levels of TFs *Ha-MYB02, Ha-NF-YB3* and *Ha-WRKY01* did not change during leaf development (Figure 3B).

**Figure 4:**
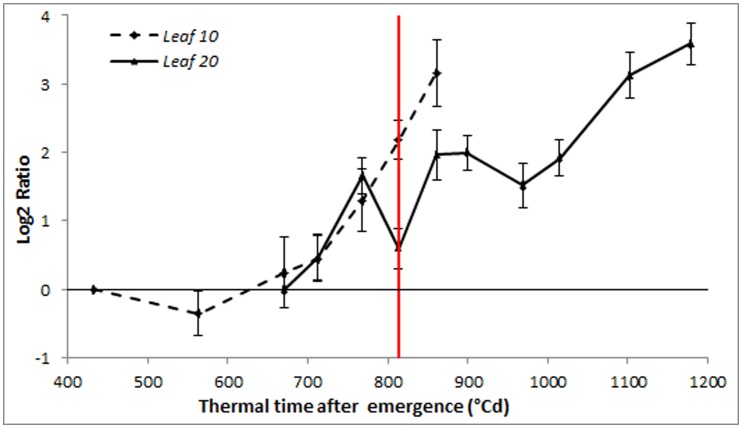
*Ha-NAC01* expression profile. Relative *Ha-NAC01* transcript level at each sampled point in relation to the level at the first sampling point determined by qPCR in leaf 10 and 20 (*Ha-EF1α* as RG). The red line indicates anthesis time. Error bars correspond to standard errors.

**Figure 5:**
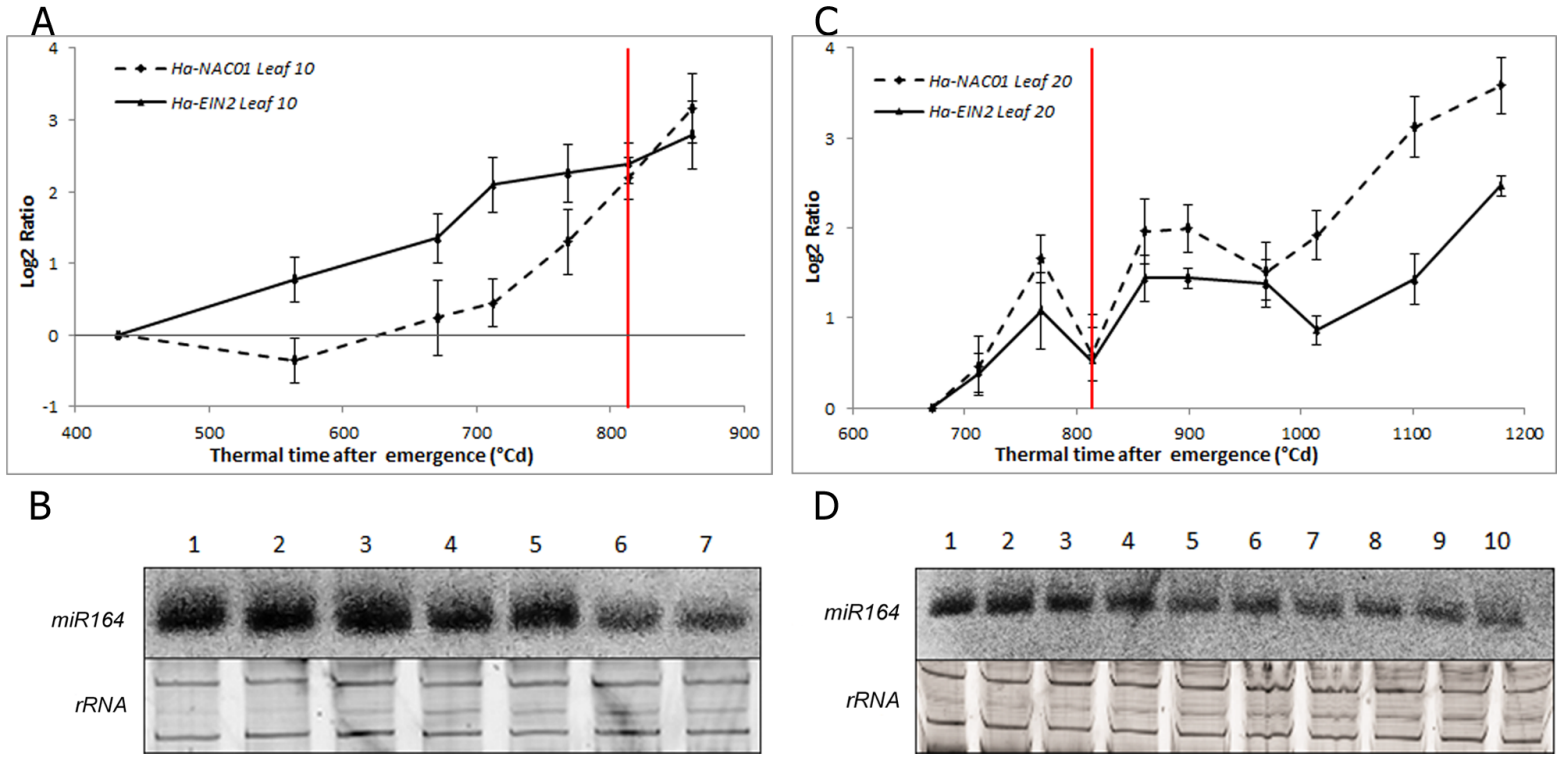
*Ha-EIN2, Ha-NAC01* and *miR164* expression profiles. (A) and (C) Transcript levels of *Ha-EIN2* and *Ha-NAC01* in relation to the first sampling point, using *Ha-EF1α* as RG in leaf 10 and 20 respectively. The red line indicates anthesis time. (B) and (D) Northern blot analysis of *miR164* levels in leaf 10 and 20, RNA from leaves 10 and 20 with an accumulation of degree days (°Cd) between 432 to 861 (B: lanes 1–7) and 670 to 1180 (D:1–10), respectively, was blotted to nylon membrane and hybridized to radioactively labelled sunflower microRNA164 probe. rRNA bands stained with ethidium bromide are shown as loading control. Error bars correspond to standard errors.

Arabidopsis *ORE1* and *EIN2* have been reported as triggers of senescence [38], [39]. Therefore, we measured the expression of *Ha-NAC01*, the putative sunflower ortholog of Arabidopsis *ORE1*, in both leaves and found that it progressively increased from emergence to approximately 700°Cd for leaf 10 and to 1,000°Cd for leaf 20, with a strong increase in the expression in the last phases of development in both leaves (Figure 4). *Ha-EIN2*, which shows high sequence similarity to Arabidopsis *EIN2*, showed different expression profiles in the two leaves. In leaf 10, *Ha-EIN2* transcript levels increased strongly in early developmental stages, even earlier than *Ha-NAC01* and prior to anthesis. By contrast, in leaf 20, *Ha-EIN2* transcript levels showed a mild and constant increase, with a significant increase towards the last stages of leaf development (Figure 5A and C).

To assess the abundance of *miR164*, previously reported as a negative regulator of *ORE1* in Arabidopsis [38], we measured its abundance by Northern blot. The abundance of *miR164* changed inversely with the expression of *Ha-NAC01* and *Ha-EIN2* in leaf 10 and 20 (Figure 5B and D). The young leaves had low levels of *Ha-NAC01* transcript levels and high levels of *miR164*; as leaf development progressed, the amount of *miR164* gradually decreased and *Ha-NAC01* expression increased.

**Figure 6:**
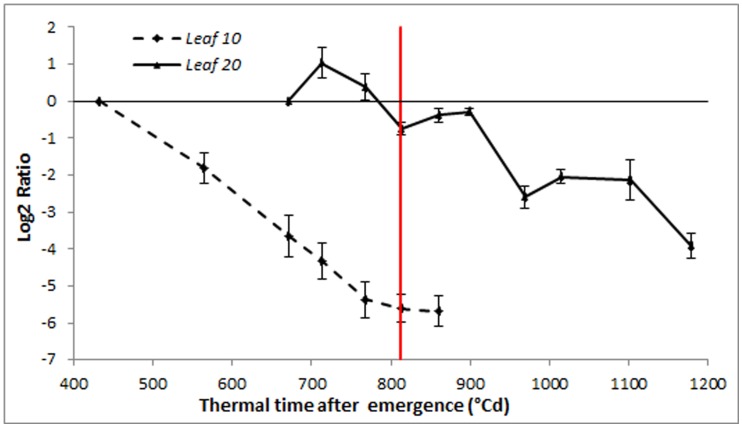
*Ha-CAB2* expression profile. Relative values of *Ha-CAB2* transcript levels referred to first sampling point, determined by qPCR (leaf 10 and 20) (*Ha-EF1α* as RG). The red line indicates anthesis time. Error bars correspond to standard errors.

Finally, we also measured the transcript levels of *Ha-CAB2* (chlorophyll a/b-binding protein) [75] in the same samples, to follow the progress of senescence at the molecular level (Figure 6). *Ha-CAB2* expression decreased during leaf development, with a steeper decrease in leaf 10 than in leaf 20. Hence, these results were consistent with those observed for physiological parameters, as expected (Figure 2).

## Discussion

The complex and highly coordinated mechanism of plant senescence may have substantial effects on agricultural production. In sunflower, as in many other crops, leaf senescence triggers abruptly, coinciding with adverse environmental conditions and foliar diseases, and limiting production. The onset and progression of senescence involves global changes in gene expression, regulated by internal and external factors. Multiple pathways respond to various stimuli and interconnect, leading to a complex regulatory network for senescence [76].

In this work, we quantified the transcription profiles of eleven candidate genes previously reported as potential regulators of senescence in the model plant *A. thaliana*. We evaluated these transcripts in association with physiological parameters, quantifying transcript levels over time in senescing sunflower leaves, to monitor the natural progression of senescence in this crop.

We found that *Ha-NAC02, Ha-NAC03* and *Ha-NAC05*, which are highly similar to Arabidopsis *ANAC072*, *ANAC055* and *ANAC019*, respectively, showed contrasting expression profiles. In Arabidopsis, *ANAC072*, *ANAC055* and *ANAC019* belong to the same clade of NAC genes and have overlapping expression patterns [77]. In sunflower, the expression of *Ha-NAC03* and *Ha-NAC05* rapidly increased toward anthesis, but *Ha-NAC02* showed an opposite expression pattern. These results suggest that different mechanisms might regulate *Ha-NAC02*, compared to *Ha-NAC03* and *Ha-NAC05*. In addition, expression analysis of the *anac019* and *anac055* mutants during senescence also indicated involvement of different signalling pathways for these genes [77].

We also found that *Ha-NAC04*, which is highly similar to Arabidopsis *ANAC047*, was up-regulated during leaf development in sunflower. In Arabidopsis, *ANAC047* was up-regulated during leaf senescence and down-regulated in mutants with defective jasmonic acid, salicylic acid or ethylene pathways, indicating a putative role for this protein during leaf senescence related to hormone signalling [78].

The Arabidopsis TF *MYB62* functions in the response to phosphate starvation and affects gibberellic acid (GA) biosynthesis. Overexpression of *MYB62* results in a typical GA-deficient phenotype, with reduced apical dominance, delayed flowering and late senescence, suggesting that MYB62 acts as a transcriptional repressor of GA biosynthetic genes [79]. In sunflower, we detected up-regulation of *Ha-MYB01* (highly similar to Arabidopsis *MYB62*) starting close to anthesis, at 700°Cd after emergence and increasing thereafter, in a period likely related to nutrient starvation, concomitant with the critical period of grain filling.

In contrast to *Ha-MYB01*, we found that *Ha-RAV01* showed a similar expression pattern to Arabidopsis *RAV1*, which increased in expression at an early stage, before the appearance of senescence symptoms and started to decrease towards the last senescence stages. Woo et al. [75] showed that RAV1 plays a regulatory role during the initiation of leaf senescence and suggested that it might control senescence by the transcriptional activation and/or repression of genes involved in the execution of leaf senescence. *RAV1* and *RAV2* were also induced by several external factors, such as pathogen attack, low temperature, drought, salt stress, darkness, and wounding [80]–[82].

In Arabidopsis, the *ORE1* transcription factor can induce leaf senescence [39], suggesting that this gene functions as a regulator of senescence. In addition, it was postulated that the micro-RNA *miR164* suppresses *ORE1* transcript levels and both elements are regulated in a loop that also involves *EIN2*, where EIN2 promotes the expression of *ORE1* and inhibits *miR164*. In this work, we evaluated the trifurcate pathway *Ha-NAC01, HaEIN2* and *miR164* [38] in two different leaves, 10 and 20 to identify differences in the expression profile associated with nutrient remobilization, during different developmental stages. We found similar expression profiles to those observed in Arabidopsis, indicating potencial conservation of this signaling pathway.

During pre-anthesis senescence, in leaf 10, the *Ha-EIN2* transcript accumulated earlier than the *Ha-NAC01* transcript, showing a significant increase in expression at the last stages, just before anthesis. At this time, the first leaf senescence symptoms appeared in leaf 10, coinciding with an increase in nutrient remobilization rate, indicating that this leaf is an important source of nutrients for the flower and younger leaves (Figure 5A). Chlorophyll and nitrogen contents in leaf 10, and (with some differences) total soluble carbohydrates, showed a rapid decrease, which was inversely proportional to *Ha-EIN2* and *Ha-NAC01* transcript accumulation. Meanwhile, mi*R164* showed high levels in the first samplings, until 700–800°Cd after emergence, and thereafter mi*R164* levels decreased towards the last stages, when *Ha-NAC01* expression increased (Figure 5A and B). *miR164* and *Ha-EIN2* showed opposite expression patterns, indicating a potential role of *Ha-EIN2* in the negative regulation of *miR164*. These results are consistent with those observed in Arabidopsis, where *miR164* negatively regulates *ORE1* and *EIN2* is up-regulated earlier in the pathway, activating *ORE1* and inhibiting *miR164* [38]. In Arabidopsis, gene expression and miRNA levels were assessed in the third and fourth foliar leaves before flowering time. In the present study, leaf 10 represents an “old leaf” from a “young sunflower plant”. Hence, this can be considered as a comparable physiological stage to those reported in Arabidopsis, showing similar transcriptional patterns for *Ha-NAC01, Ha-EIN2* and *miR164*.

*Hahb4*, a sunflower HD-Zip transcription factor previously reported as related to leaf senescence and in response to biotic and abiotic stress, is also under the control of ethylene signaling pathway [59], [83], [84]. These authors found that *Hahb-4* transcript levels were elevated in mature/senescent leaves, being its expression induced by ethylene. In addition, transient transformation of sunflower leaves demonstrated the action of Hahb-4 in the regulation of ethylene-related genes [59]. This information concomitant with the results derived from the present work, focused in the study of members of other TF families, highlight the relevance of the ethylene signaling pathways in the initiation and/or regulation of leaf senescence process in sunflower.

Opposite to leaf 10, a mid-upper leaf such as leaf 20 maintains the photosynthetic leaf area, representing an important source of photoassimilates during grain filling. When we measured post-anthesis senescence, we observed a delay in physiological symptoms for this recently expanded leaf, in comparison to leaf 10, regardless of the time of initiation on the apex (data not shown) (Figure 2). In contrast to our observations in leaf 10, *Ha-EIN2* expression increased in leaf 20, then remained stable throughout later stages with a mild increase toward the end of development. *Ha-NAC01* transcript levels in leaf 20 also increased prior to anthesis, similar to leaf 10, but then remained stable until the last stages, when its transcript level increased, before the detection of the first senescence symptoms (Figure 5C). In this leaf, chlorophyll and sugar contents remained stable during the leaf lifespan. Once *Ha-NAC01* reached high expression levels (1100-1200°Cd after emergence), these physiological parameters decreased (Figure 2A and B). Nitrogen contents showed a slow and constant decrease along leaf developmental phases (Figure 2C).

The *miR164* accumulation profile was similar to that observed in leaf 10, with a decrease of transcript levels in the last sampling when *Ha-NAC01* was up-regulated, indicating a potential role in the regulation of *Ha-NAC01* (Figure 5C and D). *miR164* levels were also opposite to *Ha-EIN2* expression, indicating a putative association between them.

These results indicate that the accumulation of Ha-EIN2 could induce expression of *Ha-NAC01* and inhibit *miR164*. In leaf 10, an earlier up-regulation of *Ha-EIN2* could lead to rapid accumulation of Ha-EIN2 protein, but in leaf 20, *Ha-EIN2* transcript levels were low during the leaf's lifespan. However, *Ha-EIN2* transcripts reached high levels later in plant development. The expression of *Ha-EIN2* could be regulated by different internal factors (such as age) and external factors (such as the incidence of radiation and red/far-red ratio), indicating a complex regulatory network for senescence.

During pre-anthesis senescence, when *Ha-NAC01* expression shows a 4-fold increase compared to the first sampling (Log2 ratio = 2) at 800°Cd after emergence, chlorophyll and nitrogen contents had decreased to 61% and 65% of the initial values, respectively. By contrast, during post-anthesis senescence, *Ha-NAC01* expression reaches a 4-fold increase at 1050°C after emergence. At this point, chlorophyll and nitrogen content showed a slight decrease, to 96% and 79%, respectively, of the initial values (Figure 2). Differences in the senescence profiles of leaf 10 and 20 in sunflower might be due to the short lifespan of leaf 10 and to light quality, accelerating senescence. Thus, *Ha-NAC01* transcript accumulation evolves slower in this leaf than in leaf 20 in relation to physiological indicators. Interestingly, in leaf 20, the *Ha-NAC01* expression profile was detected as an early senescence candidate gene.

Based on our results, leaf senescence in sunflower has two markedly different molecular profiles according to the plant developmental stage: leaf senescence during the vegetative stage, prior to anthesis and leaf senescence during the reproductive period, after anthesis. As an example of pre-anthesis senescence, leaf 10 is a developed leaf in a young plant, and the onset of senescence in leaf 10 takes place near anthesis. In this stage, nutrient remobilization from senescent organs supplies developing organs like the flower and young leaves. Different levels and quality of photosynthetically active radiation (PAR) received by the leaves can affect leaf lifespan during both pre- and post-anthesis phases [31]. Shading of the upper leaves, which decreases the incidence of PAR and the red/far-red ratio, accelerates senescence.

As an example of post-anthesis senescence, leaf 20 is a young leaf on a mature plant, and its senescence is delayed until the last plant developmental stages. In this case, the remobilized nutrients are mainly delivered to the grains. The greater incidence and quality of PAR received in this mid-upper leaf leads to a high photosynthetic activity and, consequently, to a delay of senescence, as detected by physiological measurements (Figure 2A, B and C).

In conclusion, this work identified and characterized, for the first time in cultivated sunflower, senescence-associated candidate genes previously reported in model species. Most of the putative NAC TFs transcripts evaluated, as well as *Ha-MyB1, Ha-RAV01* and *Ha-EIN2*, exhibited an early up-regulation, with increased transcript levels detected before the first senescence symptoms appeared. Interestingly, *Ha-NAC02* showed an opposite profile, with a sharp decrease in transcript levels at a very early stage of plant development. In addition, *Ha-NAC01* expression showed an inverse relationship with *miR164*, showing high expression levels before the maximum rate of decreases of chlorophyll, carbohydrate, and nitrogen content, mainly in leaf 20, suggesting that these TFs may play a role as potential triggers of senescence in sunflower plants. However, this study also showed differences of sunflower candidate genes with the Arabidopsis model, since the plant developmental stage strongly affects SAG transcriptional profiles in sunflower. In particular, *Ha-NAC01* showed an earlier up-regulation in leaves of plants close to maturity compared to younger plants.

These results allowed us to detect early-induced candidate genes. However, further functional validation will help us to clarify their role in sunflower senescence. On-going integrated analyses using the sunflower oligonucleotide expression 4×44 K Agilent chip [15] as well as functional studies involving the isolation of full-length sequences of the candidate gene, their overexpression and/or silencing in transgenic plants, both model species and sunflower, will produce new evidence to support our present results. This work opens new avenues for further studies leading to decipher metabolic pathways involved in the triggering or progression of senescence, as the same leaves were measured over the course of the full crop season to obtain physiological and molecular candidates under field conditions. To date, only a few transcriptomic studies have been published describing assays under field conditions [85], [86].

Taking these results together, our work provides a new and integrated contribution to the current knowledge of senescence in sunflower, allowing the identification of early candidate genes associated with senescence opening new avenues to explore senescence in this strategic oil crop.

## Supporting Information

Table S1 **BlastX results and Transcription Factors expression levels derived from a customized 4×44 K microarray analysis conducted on Agilent platform** [15] **.** Three different developmental stages and growing conditions were assessed (T1 = 630°Cd, T2 = 860°Cd and T3 = 970°Cd after emergence).
(XLSX)

Table S2 **BlastX results and Transcription Factor domain detected using Conserved Domain Database (CDD) (** http://www.ncbi.nlm.nih.gov/cdd **).**
(XLSX)
